# Prediction model for unfavorable treatment outcome for complicated sever acute malnutrition (SAM) in under five children admitted in hospitals at Amhara Region

**DOI:** 10.3389/fnut.2025.1523975

**Published:** 2025-02-10

**Authors:** Almaw Genet Yeshiwas, Zelalem Alamrew Anteneh, Tilahun Degu Tsega, Ahmed Fentaw Ahmed, Chalachew Yenew

**Affiliations:** ^1^Department of Environmental Health, College of Medicine and Health Sciences, Injibara University, Injibara, Ethiopia; ^2^Departments of Epidemiology, School of Public Health, Bahir Dar University, Bahir Dar, Ethiopia; ^3^Department of Public Health, College of Medicine and Health Sciences, Injibara University, Injibara, Ethiopia; ^4^Department of Environmental Health Sciences, Public Health, College of Health Sciences, Debre Tabor University, Debre Tabor, Ethiopia

**Keywords:** Amhara Region, Ethiopia, Sam, prediction, unfavorable treatment outcome

## Abstract

**Background:**

Severe acute malnutrition (SAM) affects 45 million children worldwide, with 14.89% of Ethiopian children under five suffering from it. This study validates a prediction model and develops risk scores for unfavorable treatment outcomes in SAM patients, addressing the scarcity of risk assessment tools in low-income settings and providing clinicians with a practical tool to improve decision-making.

**Methods:**

A cohort study was conducted among 915 SAM children hospitalized with SAM hospitals in Amhara Region. Data analysis was conducted using STATA 17 and R 4.4.1. A lasso-selected multivariable model developed a nomogram for clinical utility. Model performance was assessed via AUC, calibration plot and validated with bootstrapping. Decision curve analysis evaluated the model’s clinical and public health utility.

**Results:**

The incidence of unfavorable treatment outcomes of SAM cases was 27.8% (95% CI: 25, 31). Majority of admitted children in stabilization center were complicated Severe Acute Malnutrition (cSAM) under-five children a magnitude of 89.52% (95% CI: 80.5–99.82). The developed nomogram comprised seven predictors: baseline Oedema, Diarrhea, CBC test results (Anemia), Pneumonia, Folic Acid supplementation, Vitamin A supplementation and IV antibiotic treatment. The AUC of the original model was 91.3% (95% CI: 89.0, 93.5), whereas the risk score model produced prediction accuracy of an AUC of 90.86 (95% CI: 88.6, 92.9). It was internally validated by bootstrapping method, and it has a relatively corrected discriminatory performance. Decision curve analysis indicated a higher net benefit compared to treating all or none, especially for threshold probabilities above 21%.

**Conclusion:**

Our model and risk score demonstrate excellent discrimination and calibration, with minimal accuracy loss from the original, ensuring robust predictive performance. The models can have the potential to improve care and treatment outcomes in the clinical settings. Healthcare professionals prioritize the management of cSAM cases in children, particularly those presenting with baseline edema and co-morbidities such as pneumonia, anemia and diarrhea. Emphasis should be placed on timely interventions, including the administration of folic acid and Vitamin A supplementation, as well as intravenous antibiotics. Implementing a comprehensive care plan that addresses these factors will significantly improve treatment outcomes and enhance recovery in this vulnerable population.

## Introduction

The World Health Organization (WHO) defines malnutrition as inadequate or excessive consumption of nutrients, a lack of balance in essential nutrients, or difficulties in utilizing nutrients effectively (1). Complicated severe acute malnutrition (cSAM) describes an advanced stage of malnutrition where individuals show severe wasting (marasmus) or nutritional edema (kwashiorkor), along with additional medical problems like hypothermia, hypoglycemia, dehydration, severe infections, or other significant health concerns (2). SAM can also be complicated and uncomplicated based on the presence or absence of clinical features of infection or metabolic disturbance, severe demand/or poor appetite (3). The second Sustainable Development Goal (SDG2) aims to eradicate hunger, enhance nutrition, and promote sustainable agriculture. SAM directly affects nutrition, especially in children, hindering SDG2 progress. Proper management is crucial to prevent lasting health consequences (4).

Complicated severe acute malnutrition (SAM) among under-5 children is a critical public health issue that poses significant risks to their health and development, (5). This condition is characterized by severe weight loss, edema, and other life-threatening complications (6). Children with complicated SAM often require intensive medical care, including therapeutic feeding and management of associated infections (7). Early identification and treatment are essential to improve outcomes and reduce mortality rates in this vulnerable population (8).

According to the World Health Organization, approximately 45 million children under the age of five are impacted by malnutrition, with 7.3 million children receiving treatment for severe acute malnutrition (SAM) (1). Around 9% of children in sub-Saharan Africa and 15% in South Asia experience moderate acute malnutrition, while about 2% of children residing in developing nations endure severe acute malnutrition (9). While predominantly recognized as a substantial public health issue in low-income nations, malnutrition significantly contributes to the mortality rate of children under the age of 5. In 2020, severe acute malnutrition (SAM) was accountable for 5 million deaths, with an estimated 45% of all child deaths being attributed to malnutrition (10, 11).

Ethiopia ranks fifth globally in terms of malnutrition rates (12). Among children under the age of five in Ethiopia, the prevalence of severe malnutrition stands at 14.89%. This prevalence varies across regions, with rates ranging from 4.58% in Addis Ababa to as high as 25.81% in the Afar region (13).

Various factors contribute to child malnutrition. These factors in developing nations can be categorized into maternal, dietary and socio-environmental, and economic factors (14). Several studies indicate that factors linked to malnutrition include age below 24 months and a low monthly family income, maternal education, residence, along with low birth weight, diarrhea episodes, developmental delays, insufficient antenatal visits, faltering growth, and failure to deworm children (15–21).

Severe acute malnutrition (SAM) leads to severe consequences due to insufficient intake of energy, fat, protein, and essential nutrients (22). These deficiencies can impair reproductive health, hinder physical performance, and negatively affect mental well-being, increasing vulnerability to infections and diseases (23). Recent evidence also indicates that SAM is associated with long-term cognitive impairment, emotional disturbances, and delayed growth, as well as social challenges (24). Addressing these nutritional deficiencies is crucial to mitigate the extensive health impacts of SAM on individuals, particularly in vulnerable populations (25).

Children with SAM face a nine to eleven times greater risk of morbidity and mortality risks compared to healthy peers (26). Ending childhood mortality and achieving zero hunger are critical global priorities that nations strive to fulfill (27, 28). Ethiopia is actively working on these agendas by managing Severe Acute Malnutrition (SAM) in community and stabilization centers (SC) and adhering to standard treatment protocols (29). Despite these efforts, the mortality rate among treated children remains alarmingly high (30). Merely admitting SAM children to SCs does not ensure successful treatment outcomes (31). It is essential for clinicians to prioritize children at higher risk of mortality to enhance care, improve treatment prognosis, and ultimately reduce mortality risks. Admitting SAM children to stabilization centers alone does not ensure positive outcomes. Clinicians must prioritize those at higher mortality risk to enhance care, improve treatment prognosis, and reduce mortality rates.

Despite advances in the treatment of SAM, identifying children at risk of unfavorable treatment outcomes remains a significant challenge. The absence of reliable prognostic tools tailored to the specific needs of children with cSAM hampers the timely identification of high-risk individuals and the implementation of targeted interventions. Consequently, healthcare providers are often compelled to rely on subjective clinical judgment, leading to inconsistencies in risk stratification and suboptimal allocation of resources.

Moreover, the lack of standardized criteria for defining and classifying treatment outcomes in the context of cSAM complicates efforts to assess the effectiveness of interventions and compare outcomes across different settings. This variability hinders the synthesis of evidence and the formulation of evidence-based guidelines, further perpetuating the cycle of suboptimal care and poor outcomes.

The development and validation of a risk prediction model for unfavorable treatment outcomes of cSAM are urgently warranted to address the aforementioned gaps in clinical practice and research. By harnessing the power of clinical data and statistical modeling techniques, such a model has the potential to enhance risk stratification, facilitate early intervention, and improve overall outcomes for children with cSAM.

Furthermore, a validated risk prediction model can serve as a valuable tool for healthcare providers, enabling them to make informed decisions regarding the intensity and duration of treatment, the need for specialized care, and the allocation of scarce resources. By identifying high-risk individuals early in the course of illness, the model can facilitate targeted interventions aimed at mitigating complications, reducing morbidity and mortality, and optimizing resource utilization. Therefore, this study will be conducted with the specific objectives of Development and validation of risk prediction model for unfavorable treatment outcome of cSAM among under five children admitted at Hospitals at Amhara Region.

## Methods

### Study design, period and study setting

An institutional based retrospective follow-up study was conducted in hospitals found in the Amhara Region, from *August* 1, 2023 to *August 2*, 2024. In Amhara Region there is 100 public hospitals (eight comprehensively specialized, 20 generals, and 72 primary) served for 23, 558, 385 population. All hospital has separate room pediatric ward as used as a treatment center for SAM children. Health personnel follow an updated and a standardized form of treatment protocol of SAM guideline.

### Source and study population

Records of all children age 0–59 years admitted stabilization center hospitals in Amhara Region were taken as the source population, whereas all under-five children admitted with cSAM to stabilization centers of in the selected hospitals considered as the study population.

### Inclusion and exclusion criteria

All complicated under-five children with SAM admitted to therapeutic feeding unit (TFU) at, the selected hospital. Children who were admitted multiple times were only assessed for the most recent admission. However, incomplete records of under-5 children at therapeutic feeding program registration logbook with incomplete data (such as treatment outcomes, age, sex date of admission and discharge) were excluded from the study.

### Sample size determination

The minimum sample size that represents the source population was ensuring to consider the number of events per parameter (EPP) of ≥10, (32). By using the following formula EPP = $\frac{n\phi }{p}$ , Where EPP is the number of events per parameter, n is the sample size, 𝜙 is the overall rate of the outcome and P is the number of parameters to be involved in the model development (32). Sample size estimation of the study followed the following assumptions using is the most frequently used conventional rule of thumb formula for minimizing the problem of overfitting in multivariable prediction modelling, Using EPP of ≥10, 21 parameters, and 𝜙 of (0.3787) which was taken from the previous studies (33) and design effect of (1.5). Therefore, the final adequate sample size considering a 10% non-response rate was 915, under-five children admitted to stabilization centers in selected hospitals within the Amhara Region.

### Sampling technique

A multistage stratified random sampling method was used to select children with SAM in hospitals across the Amhara Region. From a total of 28 general and comprehensive specialized hospitals, 9 were randomly selected. Additionally, 19 out of 69 primary hospitals were chosen, representing 28% of all hospitals in the region. The sample size was proportionally distributed among the selected hospitals and across different professional strata. Subsequently, a simple random sampling technique was applied using the serial numbers from the therapeutic feeding registration logbook of SAM children. The unique medical record numbers were then matched to the randomly selected serial numbers. Finally, the medical charts of the chosen children were retrieved from the card room for data collection.

### Variables of the study

The dependent variable was unfavorable treatment outcome (Yes/No) of under-5 children admitted for SAM. Meanwhile, the independent variables also included socio-demographic factors (age, sex of the child, place of residence), clinical conditions (loss of appetite), presence of nutritional oedema, co-morbidities (pneumonia, HIV status, diarrhoea, anaemia, malaria and tuberculosis), routine medication intake (intravenous (IV) antibiotic treatment, folic acid, vitamin A supplementation, deworming, Intake of F75 and F100) and level of anthropometric deficits at admission.

### Operational definitions

Unfavorable treatment outcome: The treatment outcome of under-5 children admitted for SAM being death, and/or treatment failure and/or non-respondent and/or defaulter, and/or staying longer time in the hospital (34).

*Length of stay*: The number of days the child stayed in the hospital from admission until death or recovered, (35).

*Severity of oedema*: was defined as grade + if involving only the feet, ++ if also involving the legs or hands, and +++ if also involving the area around the eyes (36, 37).

*Complete record*: if age in months, sex of the child, date of admission and discharge, type major complications, treatment outcome and other parameter well recorded.

*Saver acute malnutrition (SAM)*: was defined as the presence of any bilateral pitting oedema (kwashiorkor), MUAC <11.5 cm (marasmus) or weight-for-height or length Z-scores < −3 (marasmus), or Marasmic-kwashiorkor (1, 38).

*Complicated SAM*: Children (6–59 months) who are acutely malnourished with associated medical complications and/or poor appetite; and infants less than 6 months with SAM, need to be treated in an inpatient care facility until they are well enough to continue nutritional rehabilitation (39).

*Co-morbidities*: In children with severe acute malnutrition, having TB and/or HIV and/or malaria and/or pneumonia, and/ or diarrhoea, and/or severe anaemia co-infection on admission to stabilization center.

*Stabilization phase*: children with cSAM are initially admitted to an inpatient facility for stabilization. In this phase: Life-threatening medical complications are treated, Routine drugs are given to correct specific deficiencies, Feeding with F-75 milk (low caloric and sodium) is begun (39).

Anaemia was defined with a haemoglobin level below 11 gm/dl (haematocrit level < 33%) at admission.

*Severe Anaemia*: If the haemoglobin concentration is less than 40 g/L or the packed–cell volume is <12% the child has very severe anaemia.

*Rehabilitation Phase*: Children that progress through phase 1 and transition phase enter phase 2 (rehabilitation phase) when they have a good appetite and no major medical complication. In this phase: Routine drugs are continued, Feeding with RUTF or F100 is started (40).

*Cure from SAM*: was achieved when weight-for height/length is ≥ − 2 Z-scores and they have had no oedema for at least 2 weeks, or MUAC is ≥12.5 cm, and they have had no oedema for at least 2 weeks without any acute medical complications (39).

*Recovered*: is defined as when the child is treated for acute medical complications at a stabilization center and transferred to OTP (not cured yet) for continuous SAM treatment.

## Patient and public involvement

None or there is no involvement of patients and/or the SAM cases in the design, or conduct, or reporting or dissemination plans of this research.

### Data collection tools and procedures

A structured data extracting tool (checklist) was developed using different literature. Predictors of unfavorable treatment outcomes such as sociodemographic factors, clinical-related factors, medical-related factors, comorbidities, and anthropometric related factors were extracted. Therapeutic feeding program registration logbook and under-5 IMNCI registration were used for data extraction. The Data were collected by 6 trained Nurse and two master’s holder health professionals were recruited to manage the data collection processes. Data quality was managed by training and appropriate supervision of data collectors. Overall supervision was made by the supervisor and principal investigator. The collected data were checked for completeness, clarity, and accuracy. The quality checking was done daily after data collection and correction was made before the next data collection measures.

### Data processing and analysis

The data are collected through KoboCollect data collection toolbox, and then it was exported to STATA version 17 and RStudio version 4.3.3 statistical software for analysis. Variables with missing data was managed by multiple imputation techniques using the “mice” package in R-software assuming that the data were missed at random (MAR). The imputation process was performed in the whole dataset and 5 imputed datasets were generated. Sensitivity analysis was done to investigate the plausibility of the MAR assumption. Descriptive statistics, frequencies, and percentages were done for categorical variables. The normality distribution test was done using the Kolmogorov–Smirnov test. Incidence was calculated to determine the occurrence of unfavorable treatment outcomes.

For the multivariable prediction model development: The theoretical design of the incidence of unfavorable treatment outcome at a future time “t” is a function of prognostic determinants like demographic factors, clinical factors, and behavioral factors measured or ascertained at one or more time points before the occurrence of the unfavorable treatment outcome, i.e., “t_0_” which is the moment of prognostication.

### Model development and validation

The Least Absolute Shrinkage and Selection Operator (LASSO) algorithm was used to select the most potent predictors. Penalized regression method was preferred for feature selection to develop unbiased and most parsimonies unfavorable treatment outcome risk prediction model by minimizing overfitting (41).

Variables were considered for multivariant unfavorable treatment outcome prediction model development based on their easily obtainability, biologically plausible relationship with the outcome, and ease of interpretation in clinical practice. The lasso model with optimum shrinkage factor and minimum cross-validation mean deviance was selected to take predictors with non-zero coefficients for multivariable logistic regression analysis.

The most potent predictors selected by LASSO regression were incorporated into the multivariable analysis. Then, variables were removed from the multivariable model step by step to build a simplified reduced model at a significance level of *p*-value<0.15 just to be more liberal. The final simplified risk prediction model was presented in the form of a nomogram and its performance was described by assessing its discriminatory power and calibration. The discriminatory power of the simplified risk prediction model was determined by calculating c-statistics. The c-statistics might range from 0.5 (no predictive ability) to 1 (perfect discrimination) (42, 43). The developed risk prediction model was also assessed qualitatively by using Swets’s criteria, which values range from 0.5–0.6 (bad), 0.6–0.7 (poor), 0.7–0.8 (satisfactory), 0.8–0.9 (good), and 0.9–1.0 (excellent) (44).

The calibration of the model was presented graphically using the calibration plot and Hosmer-Lemeshow test. For the model calibration test, a value of *p* > 0.05 suggested a good model calibration. The prediction performance of the model was also assessed by a prediction density plot. Bootstrap resampling (with 1,000 repetitions) of the original set was performed for internal model validation to calculate a relatively corrected C-statistics (AUC). Patients were classified as high and low risk of unfavorable treatment outcomes using the optimal cut-off point identified by the Youden index. The traditional risk prediction model performance measures (discrimination and calibration) could not address the issue of how useful the developed nomogram would be in clinical practice. Therefore, Decision Curve Analysis (DCA) was done to provide a clinically interpretable risk prediction model which could show the clinical and public health impact of using it. It was plotted by putting the net benefit of carrying out a specified intervention using the developed risk prediction model or intervention on all of the patients or none of them in the y-axis and the threshold probabilities in the x-axis. The study was reported per the TRIPOD (transparent reporting of a multivariable prediction model for individual prognosis or diagnosis) statement (45).

## Result

From April 29,2020 till march 31, 2024 a total of 864 admitted SAM under five children who had complete information were involved in this study. The rest 51 (25 of them the outcome is not recorded and 26 were under treatment) SAM under five children were not included in the study. Therefore, the overall completeness of SAM under five children enrolled in stabilization centers was 94.4%.

### Socio-demographic and admission characteristics

The majority 493 (57.1%) admitted SAM cases in stabilization centers were came from rural area. Similarly, 491(56.8%) admitted SAM under five children were male. The median (±IQR) age of a children were 11 (±14) years old. On the other hand, 238 (27.5%) of admitted SAM cases in stabilization centers were had oedematose. Beside of this the complete blood count (CBC) result verified that 239(27.7%) of SAM case were had Anemia. Adjacent to this 147(17%) of complicated SAM case were develop Pneumonia. From the total cases above 2/3^rd^ (86.5%) were marasmus clinical characteristics (Table 1).

**Table 1:** Baseline socio-demographic, comorbidity and routine medication characteristics of admitted SAM cases at stabilization center at hospitals in Amhara Region August, 2023-2024 (*n* = 864).

Characteristics	Characteristics category	Frequency	Percentage (%)
Age category	<24 months	726	84.0
	> = 24 months	138	16.0
Residence	Rural	493	57.1
	Urban	371	42.9
Sex	Male	491	56.8
	Female	373	43.2
Oedema	No	626	72.5
	Yes	238	27.5
Kidney function test result	Normal	715	82.8
	Abnormal	149	17.2
Diarrhea	No	587	67.9
	Yes	277	32.1
Pneumonia	No	717	83.0
	Yes	147	17.0
	Marasmus	747	86.5
Clinical forms of malnutrition	Kwashiorkor	15	1.7
	Marasmus-kwashiorkor	102	11.8
TB screen result	Negative	820	94.9
	Positive	44	5.1
HIV/AIDS test result	Negative	841	97.3
	Positive	23	2.7
Length of stay	< 15 days	730	84.5
	> = 15 days	134	15.5
CBC test result	Presence of Anemia	239	27.7
	No Anemia	625	72.3
Supplementation of folic acid	No	104	12.0
	Yes	760	88.0
Supplementation of Vitamin-A	No	601	69.6
	Yes	263	30.4
Deworming	No	299	34.6
	Yes	565	65.4
Excusive breast feeding	No	314	36.3
	Yes	550	63.7
IV antibiotic treatment	No	156	18.1
	Yes	708	81.9
Intake of F75	No	46	5.3
	Yes	818	94.7
Intake of F100	No	46	5.3
	Yes	818	94.7
History of bottle feeding	No	70	8.1
	Yes	794	91.9

IV, intravenous, TB, Tuberculosis.

### Treatment outcomes

**Figure 1:**
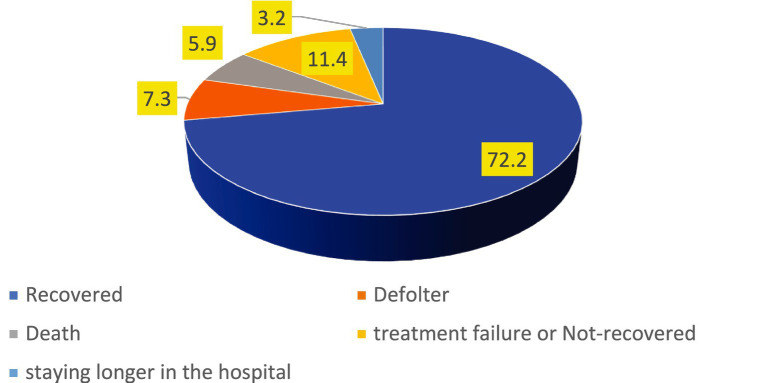
The incidence of unfavorable treatment outcome of admitted SAM cases at stabilization center in hospitals at Amhara Region August, 2023-2024 (*n* = 864).

The findings of this study revealed that the proportion of treatment failure or not-recovered, defaulter, death rates and staying longer in the hospital were 98(11.4%), 63(7.3%), 51(5.9%) and 28(3.2%) respectively. However, the overall incidence of unfavorable treatment outcome among the study participants was 27.8% (95% CI: 25, 31%) (Figure 1).

### Variable selection and model diagnosis

A total of 39 models were generated using LASSO regression with a 10-fold cross-validation selection method and LASSO estimator. The 34th model was found to be the most parsimonious model with an optimum penalty factor (lambda) of 0.008602 and minimum cross-validation mean deviance. Among 19 co-variants entered in LASSO regression, 12 potential features (predictors) were selected (Table 2).

**Table 2:** Optimum shrinkage factor (lambda) and potential predictors identified by lasso regression by the 10-fold cross-validation selection method.

ID	Description	Lambda	No. of nonzero coef.	Out-of-sample dev. Ratio	CV means deviance
1	First lambda	0.185327	0	0.0004	1.181215
33	Lambda before	0.009441	10	0.4466	0.653944
34*	Selected lambda	0.008602	10	0.4468	0.653692
35	Lambda after	0.007838	10	0.4467	0.653799
39	Last lambda	0.005402	12	0.4453	0.65548

^*^Lambda selected by cross-validation, No. of nonzero coef., number of nonzero coefficients; dev. Ratio, deviance ratio; CV, cross-validation.

The identified 10 potential predictors selected by lasso regression were incorporated into the multivariable analysis. These variables were; Oedematous, Diarrhoea, complete blood count (CBC) or Haemoglobin test result, Pneumonia, Folic acid supplementation, kidney function test, Vitamin-A supplementation, intake of F75, IV antibiotics treatment and intake of F100.

### Development of an individualized risk prediction model

An individualized unfavorable treatment outcome risk prediction model was developed based on multivariable binomial regression analysis using the identified potential predictors selected by lasso regression. Most of the predictors are easily ascertainable right at patient enrolment time. The role of each predictor was assessed by reducing them one by one from the full multivariable model at a significant level of *p*-value <0.15 (Table 3).

**Table 3:** Multivariable logistic regression analysis and model reduction using potential predictors of unfavorable treatment outcome of admitted SAM cases at stabilization center, in hospitals at Amhara Region North West Ethiopia August, 2023–2020 (*N* = 864).

Prognostic Predictors selected by lasso algorithm	Original model		Model reduction		Risk score
	Coef (95% CI)	p-value	Coef (95% CI)	p-value	
Presence of oedemaᵅ					
No	0		0		
Yes	1.373 (0.891, 1.862)	<0.001***	1.409 (0.955, 1.872)	<0.001***	4
Kidney function test					
Normal	0				
Abnormal	0.104 (−0.487, 0.684)	0.727778			
Diarrheaᵅ					
No	0		0		
Yes	1.7791 (1.334, 2.238)	<0.001***	1.803 (1.363, 2.256)	<0.001***	5
Pneumoniaᵅ					
No	0		0		
Yes	1.901 (1.349, 2.467)	<0.001***	1.958 (1.415,2.518)	<0.001***	6
CBC test resultᵅ					
Presence of Anemia	−1.569 (−2.058, −1.095)	<0.001	−1.578 [−2.025, −1.142]	<0.001***	5
No Anemia	0		0		
Folic Acid Supplementationᵅ					
No	−1.734 (−2.374, −1.112)	<0.001***	−1.787 (−2.417, −1.175)	<0.001***	5
Yes	0		0		
Vitamin-A supplementationᵅ					
No	0.357 (−0.109, 0.819)	0.131044	0.358 (−0.104, 0.818)	0.127002	1
Yes	0				
IV antibiotic treatmentᵅ					
No	−2.283 (−2.832, −1.758)	<0.001***	−2.302 (−2.848, −1.779)	<0.001***	7
Yes	0		0		
Intake of F75					
No	0.5186 (−1.377, 0.341)	0.235008			
Yes	0				
Intake of F100					
No	0.244 (−0.987, 0.515)	0.524744			
Yes	0				
Intercept	1.373 (0.891, 1.862)	0.000287 ***	1.681 (0.843, 2.542)	0.000101 ***	

Coef., coefficients; CI, confidence interval; ᵅ = Variables included in the final simplified model. *p*-value denoted with ‘*’ = <0.05, ‘**’ = < 0.01, ‘***’ = 0.001.

### Nomogram of the final model

Predictors used in the construction of the nomogram are; Baseline Oedematous, Diarrhoea, CBC test result, Pneumonia, Folic acid supplementation, Vitamin A supplementation and IV antibiotics treatment. The developed nomogram could be used to calculate the risk of individual patients for unfavorable treatment outcome easily. For instance; The risk of under-5 admitted SAM cases who had baseline oedema with co-morbidity of Pneumonia, Anemia, Diarrhea, without supplementation of folic acid, Vitamin-A supplementation and intravenous antibiotics as follows.

**Figure 2:**
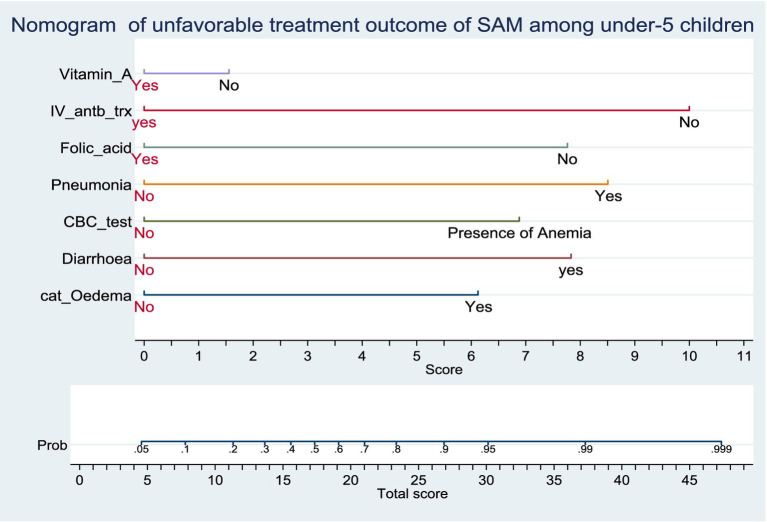
Nomogram of unfavorable treatment outcome of admitted SAM cases at stabilization center in hospitals at Amhara Region, August 2023-2024 (*n* = 864). Score = the risk score for each category of predictors, Total score = The total risk score identified by adding up the score for each predictor category of a patient, Probability of unfavorable treatment outcome = The risk probability of unfavorable treatment outcome for the identified Total score.

SAM under-5 children who had a baseline oedema and the score for this category of comorbidity is 6. The SAM case who had Pneumonia and the score for this category is 8.5. The other co-morbidity SAM case was Anemia and Diarrhea and its score for this category is 7 and 8, respectively. On the other hand, admitted SAM cases get folic acid and Vitamin-A supplementation and IV antibiotic treatment and its score for this category is zero. The total score of each predictors category was 29.5. The risk of the patient for unfavorable treatment outcome with this total score would be read from the nomogram and it is 0.94 (high risk) (Figure 2).

### Performance of the nomogram developed

**Figure 3:**
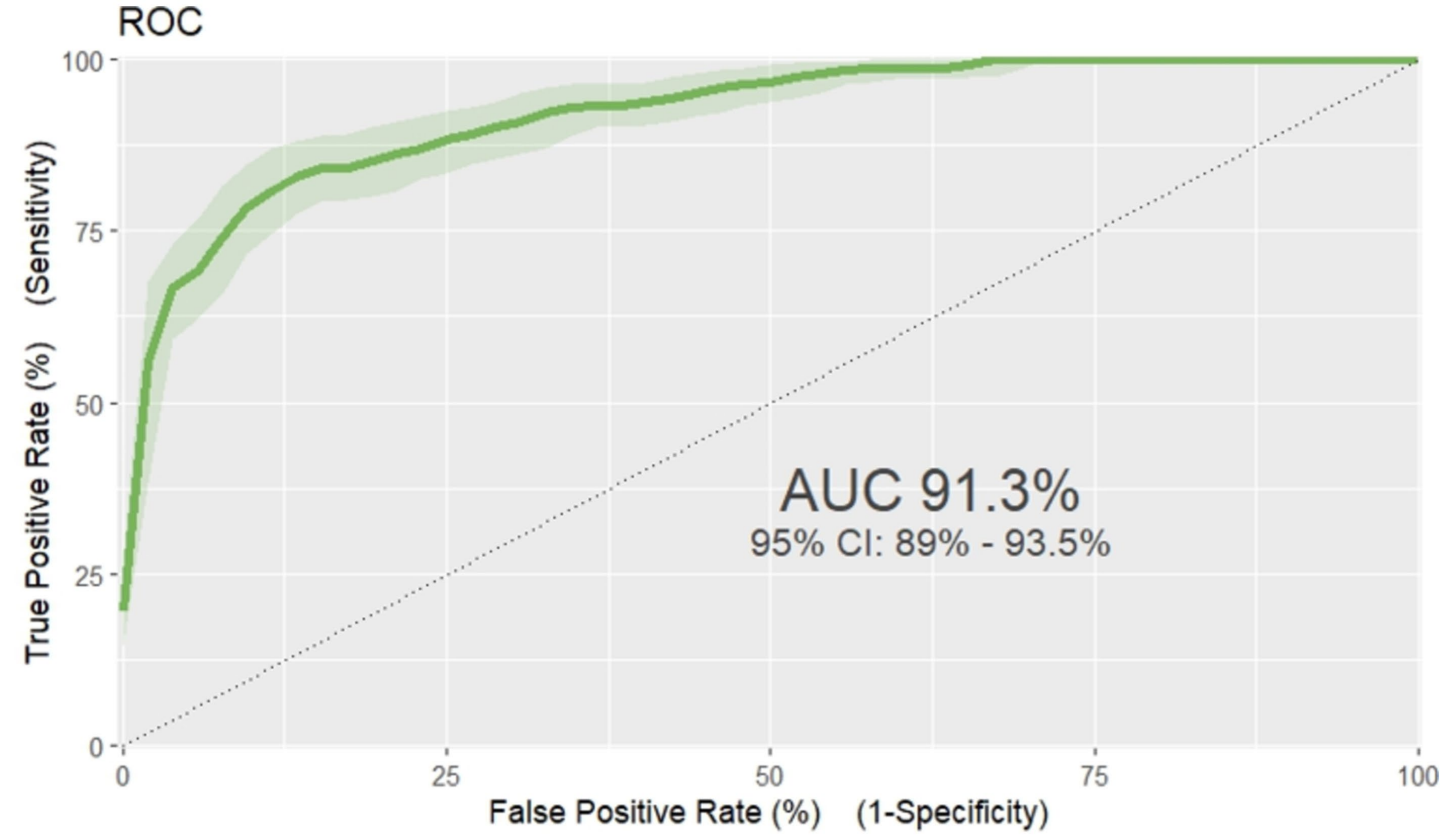
ROC curve of unfavorable treatment outcome risk prediction model of admitted SAM cases at stabilization center in hospitals at Amhara Region August, 2023-2024 (*n* = 864).

Based on the discriminatory power and calibration plot the developed nomogram performance was evaluated. The area under the curve (AUC) of receiver operating characteristics curve (ROC-curve) of the original model found to have a discriminatory power of (AUC = 0.913, 95% CI: 0.890, 0.935) (Figure 3). The prediction role of individual prognostic determinants; Baseline Oedematous (Yes), Diarrhoea (Yes), Pneumonia (Yes), CBC test result (presence of Anaemia), IV antibiotics treatment (No), Folic acid supplementation (No) and Vitamin-A supplementation (No) was assessed and it was found to be 0.699, 0.714, 0.674, 0.669, 0.658, 0.621 and 0.555, respectively.

**Figure 4:**
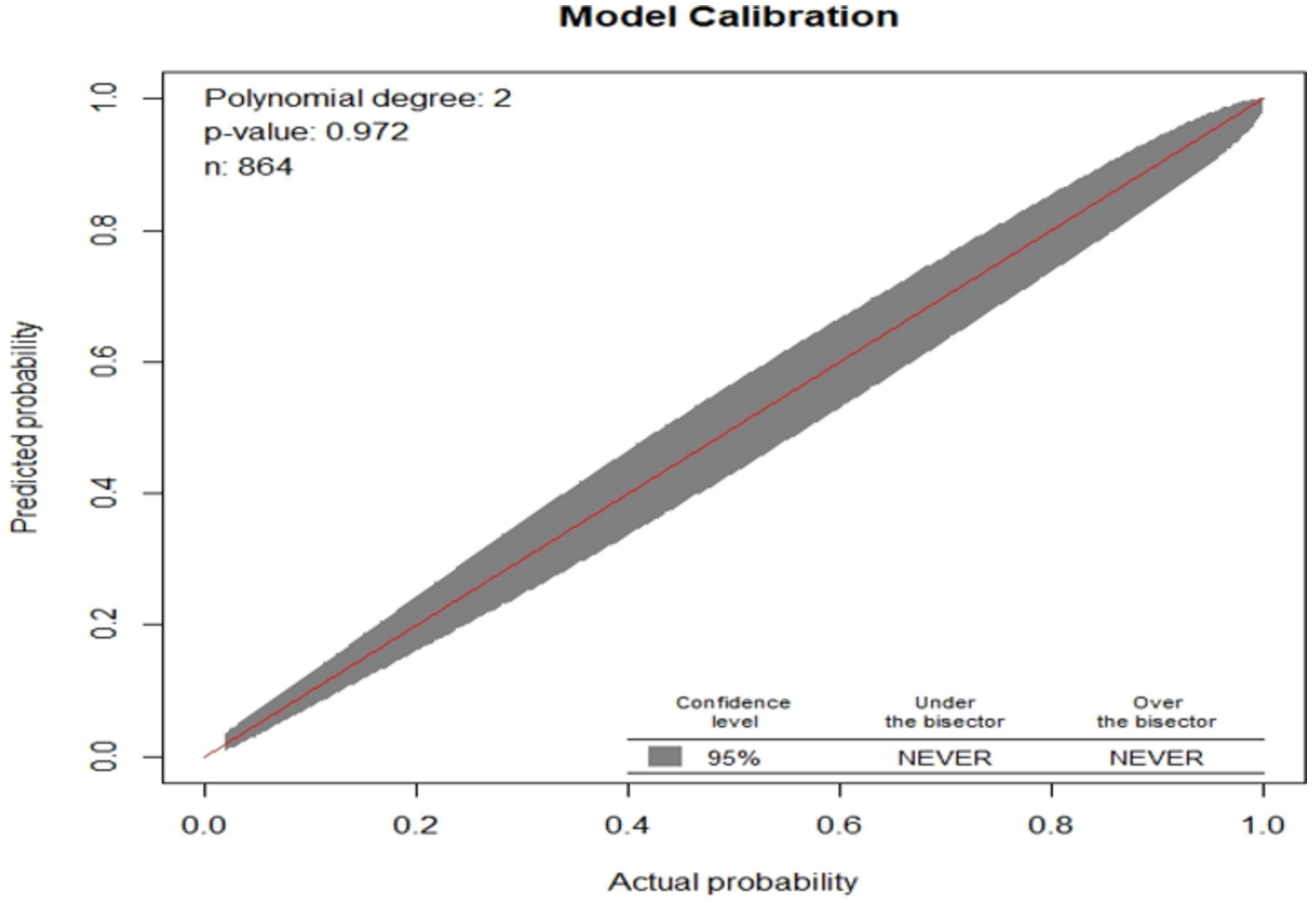
Observed versus predicted unfavorable treatment outcome probabilities of admitted SAM cases at stabilization center in hospitals at Amhara Region August, 2023-2024 (*n* = 864).

The model fitness test had a *p*-value of 0.4449 the calibration curve is nearly 45 degrees, showing that there is no difference between predicted and the observed probabilities (Figure 4).

**Figure 5:**
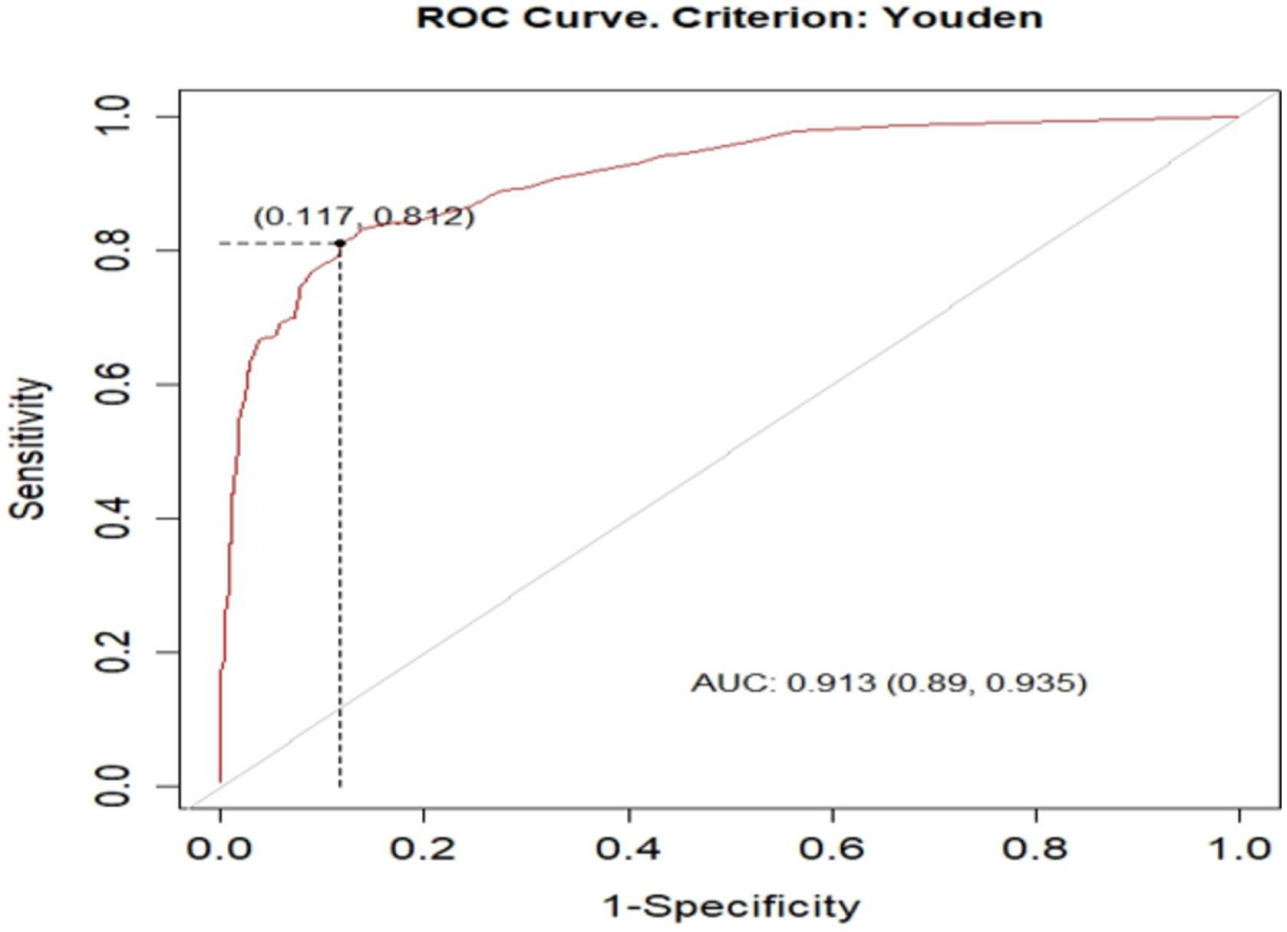
optimal cut of point (Youden index) cut off point of unfavorable treatment outcome for admitted SAM cases at stabilization center in hospitals at Amhara Region August, 2023-2024 (*n* = 864).

Based on the default 0.5 cut off probability, the original model has accuracy (ACC) of 0.872 (12.8% misclassification rate), sensitivity(S) 0.671, specificity (SP) 0.949, positive predictive value (PV+) 0.834, and negative predictive value (PV-) 0.882. However, based on the optimal cut of point (Youden index) cut off point 0.6955 probability, the model has accuracy (ACC) was 0.863 (95% CI: 0.839, 0.886), sensitivity(S) 0.813 [95% CI: 0.757, 0.859], specificity (SP) 0.883 [95% CI: 0.855, 0.907], positive predictive value (PV^+^) 0. 728 [95% CI: 0.676, 0.791] and negative predictive value (NPV^-^) 0.925[95% CI: 0.898, 0.941], (Figure 5).

**Figure 6:**
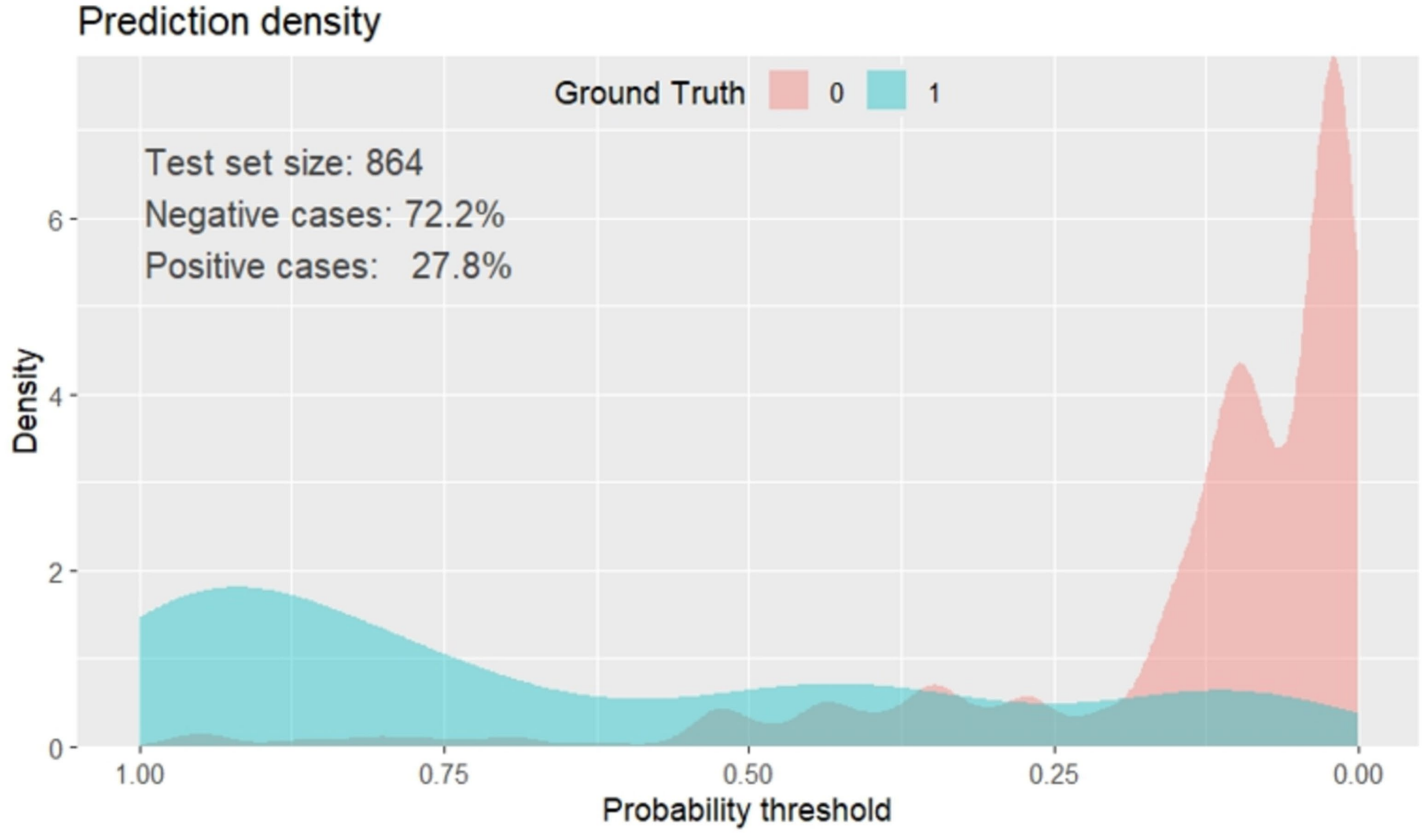
Prediction density plot of unfavorable treatment outcome of admitted SAM cases at stabilization center in hospitals at Amhara Region, August, 2023-2024 (*n* = 864).

The prediction density plot shows the extent to which the developed nomogram classified patients with the unfavorable outcome as “1” and those without unfavorable outcome as “0.” The density plot of the reduced multivariate model indicated that 27.8% of the study subjects were with poor outcomes of SAM (positive cases). The graph with red one represents children with low risk of SAM, and the blue one was children at high risk of unfavorable treatment outcome of SAM. The plot showed some overlap in the model at a limited range of threshold probabilities. It is not 100% perfect, no model is 100% perfect. Therefore, the plot below shows the general insight of the prediction density of the nomogram, (Figure 6).

### Model validation

**Figure 7:**
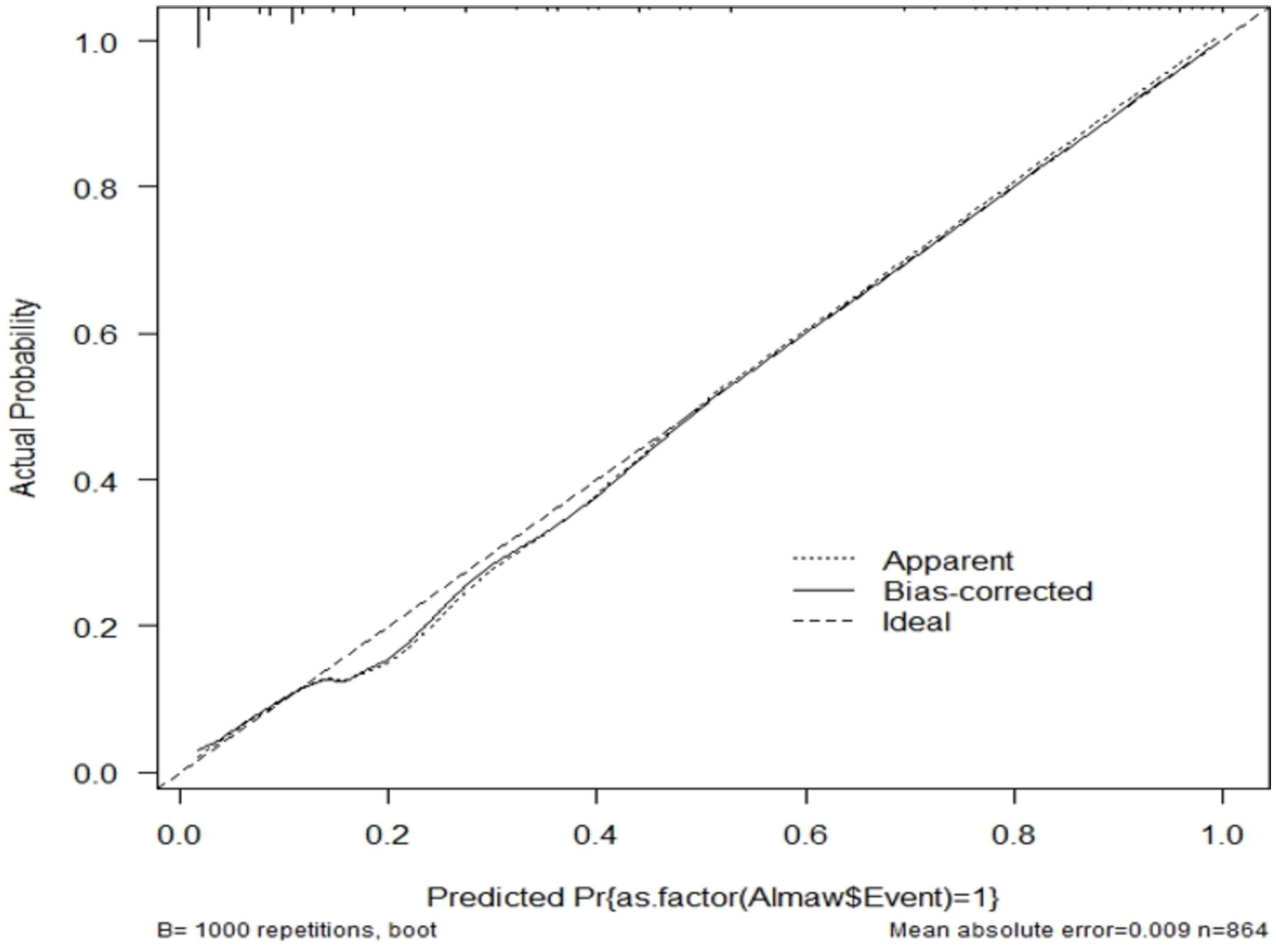
Calibration model after bootstrap for unfavorable treatment outcome of admitted SAM cases at stabilization center in hospitals at Amhara Region, August, 2023-2024 (*n* = 864).

The model was validated internally using the bootstrapping method to avoid over-interpretation and minimize too optimistic results from the original model by using “mrs” package. Bootstrap method is preferred over split-half method and cross-validation for model more stable. It was performed by drawing bootstrap samples of 1,000 repetitions with replacement and was found to have a relatively corrected discriminatory power of 0.9086 (95% CI: 0.886, 0.929). The *β* coefficients from the bootstrapped model produced marginally the same results as the original β coefficients. The optimism coefficient for the validated model was 0.008, which is minimal and ensures the less likelihood of optimism induced over fitting of the developed nomogram and its prediction capability when it is applied in external settings. The finding is also supported by the model calibration test (*p*-value 0.009), indicates a very good agreement between predicted and observed probabilities; very slightly that the apparent curve seems to outperform the bias-corrected curve between 0.18 and 0.5 predicted probabilities. Therefore, given the limited optimism, and excellent calibration, the model might perform well in a new sample (Figure 7).

To predict an individual estimated risk for unfavorable treatment outcome of cSAM based on the identified predictors of validated regression coefficients as: the estimated probability of risk for unfavorable treatment outcome = 1/1 + exp-(1.68 + 1.401) * Baseline Oedematous (Yes) + 1.80* Diarrhoea (Yes) + (−1.58) * CBC test result (presence of Anaemia) +1.96* Pneumonia (Yes) + (−1.79) * Folic acid supplementation (No) + 0.36* Vitamin A supplementation (No) + (−2.03) * IV antibiotics treatment (No).

### Decision curve analysis (DCA)

Regarding the decision to use our model, the decision curve outperforms the default strategies (referring all and none) across the entire range of threshold probabilities. The purple line represents the developed risk prediction nomogram, the thin black line represents the assumption that all patients are at risk of unfavorable treatment outcomes and the thick black line represents that none of the patients are at risk of unfavorable treatment outcomes.

The net benefit was calculated by subtracting the proportion of all patients who are false positive from the proportion who are true positive, weighting by the relative harm of not taking an intervention compared with the negative consequences of unnecessary intervention. The relative harm was calculated as; ( $\frac{\mathrm{pt}}{1-\mathrm{pt}}$ ) (46). Here, “p_t_” stands for the threshold probability, where the expected benefit of a certain intervention is equal to the expected benefit of avoiding an intervention. The DCA plot shows, the net benefit of using the model to carry out a certain intervention, which could be determined according to the status of the clinical setting, to tackle the unfavorable treatment outcome in admitted SAM cases at stabilization center among under-5 children was found to be higher than intervening on all or none of the SAM cases.

**Figure 8:**
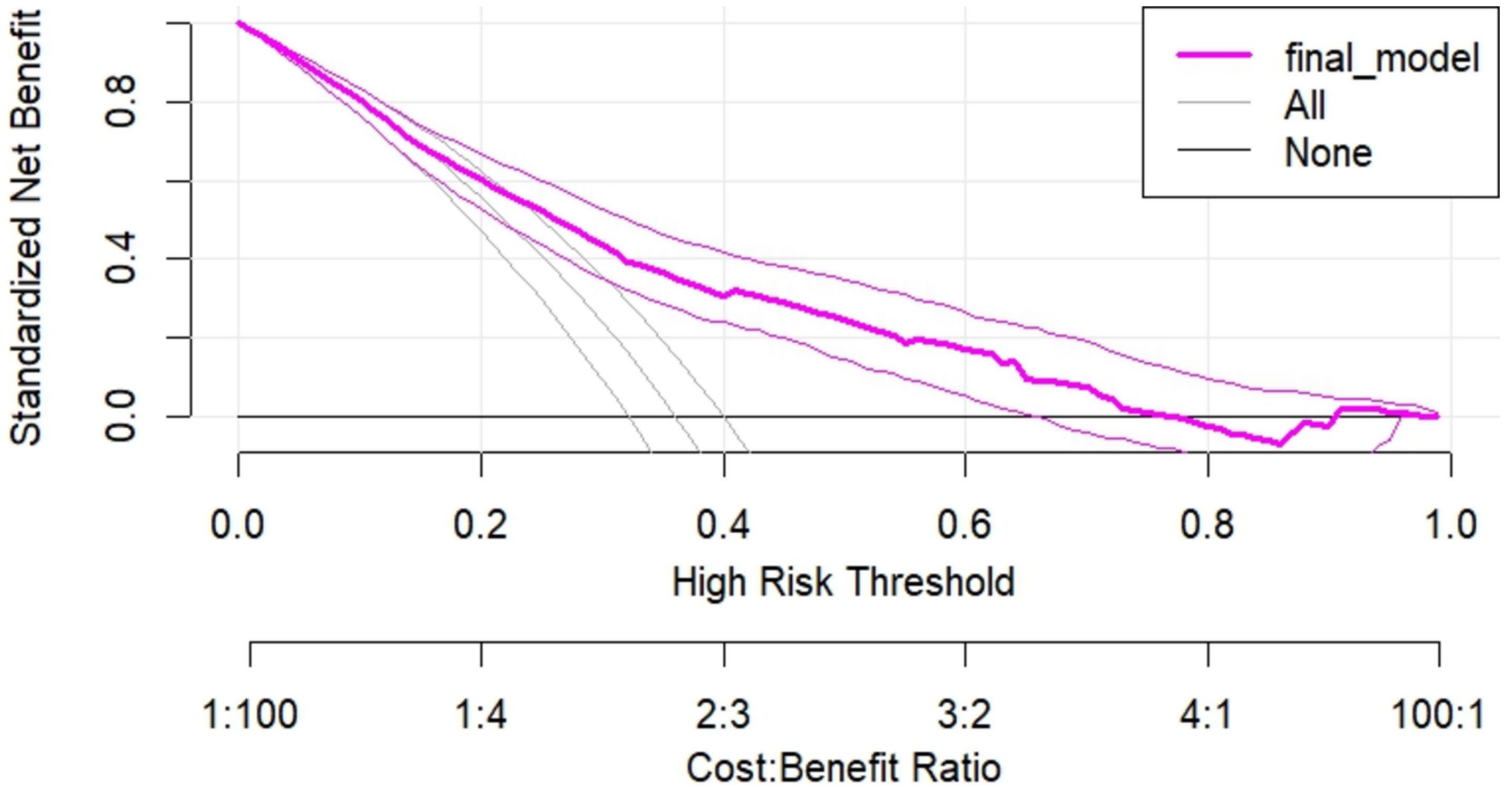
Decision curve plot showing the net benefit of the developed model for carrying out a certain intervention measure in in admitted SAM cases at stabilization center among under-5 children at risk of unfavorable treatment outcome compared to all or none schemes.

The decision curve showed that if the threshold probability is greater than 21%, using the developed risk prediction nomogram in this study to predict unfavorable treatment outcomes in admitted SAM cases at stabilization center among under-5 children adds more benefit than the intervention on all or none of the patients’ strategies. This implies that our model has the highest clinical and public health importance. Therefore, decisions made using the model such as safely discharging children with some medications or keeping children for more intensive care in the hospitals has a higher net benefit (Figure 8).

### Risk classification using a nomogram

The final simplified model was presented in the form of a nomogram just for practical utility. Patients are classified as at low, and high risk of unfavorable treatment outcomes based on the risk probability identified using the nomogram. The risk probability calculation using the nomogram is too simple that any health professional at any level can do. Hence, using the cutoff (0.6955) identified by the Youden index method, patients are classified as at low, and high risk of unfavorable treatment outcomes. The proportion of unfavorable treatment outcome in low (<0.6955), and high-risk groups (≥0.6955) were; 2.6 and 89.4%, respectively. On the other way for simple interpretation in the clinical settings, we categorized risk scores into less than twenty-one points (< 21) (low-risk group), and greater or equal to twenty-one points (≥ 21) points (high-risk group) based on Youden index (optimal cut-off point) which corresponds to the probability of 0. 6,955 in the model. Therefore, a child can have a minimum and maximum risk score of 0 and 33, respectively. The incidence of high-risk groups of unfavorable treatment outcome of SAM cases were 170(19.7%) and the rest 694(80.3%) cases were categorized in the low-risk group, (Table 4).

**Table 4:** Risk stratification for unfavorable treatment outcome of SAM admitted cases using simplified prediction score.

Risk category	Frequency	Incidence of poor outcome
Low (<21 score) or (<0.6955),	694 (80.3%)	18 (2.6%)
High (> = 21 score) or (≥0.6955)	170 (19.7%)	152 (89.4%)
Total	864	170 (19.7%)

Risk category* = the risk probability calculated using the nomogram.

## Discussion

In this study, the incidence of unfavorable treatment outcomes from SAM was 27.8% [95% CI: 25, 31%] and the treatment success rate was 72.2%, which was lower than WHO recommended success rate (>90%) (36). This finding is higher than the studies conducted in Sera lion (17%) (47). This high death rate and low recovery rate could be a result of delay at presentation to a stabilization center, the occurrence of recurrent infections, presence of co-morbidities and non-adherence (by healthcare providers) to the current SAM treatment guideline (48). However, it is lower compared to studies done in Ethiopia (21%) (49). This discrepancy might be because of the study characteristics. For instance, previous studies included both moderate and severe forms of acute malnutrition, which might potentially be associated with good treatment outcomes.

A multivariable risk prediction nomogram was developed and internally validated to predict unfavorable treatment outcomes in SAM patients. The primary goal was to create a practical tool for clinicians to improve SAM management, especially in highly burdened countries. Predictors for the model were selected based on their association with outcomes using the Lasso method, a penalized regression technique. This method reduced 19 potential predictors to 10, allowing the development of a parsimonious and robust model. These predictors were further analyzed through multivariable regression, where they were gradually removed based on their significance level (*p* < 0.15).

The final, simplified model, presented as a nomogram, included seven independent predictors of unfavorable outcomes: Baseline Oedematous status, Diarrhea, CBC test results (Anemia), Pneumonia, Folic Acid supplementation, Vitamin A supplementation, and IV antibiotic treatment. This model demonstrated strong predictive performance with an AUC of 0.913 and good calibration, indicating that predicted probabilities closely matched observed outcomes. Internal validation through bootstrap resampling (10,000 iterations) confirmed the model’s reliability, with acceptable levels of specificity, sensitivity, PPV, and NPV at a cut-off point of 0.6955, identified via the Youden index. This model offers flexibility, as the threshold can be adjusted to prioritize specificity or sensitivity, depending on clinical needs and available resources. During internal validation using the bootstrapping method, the model was trained on a bootstrap sample and tested on the original dataset. The difference between the apparent performance (in the derivation dataset) and the tested performance (in the tested dataset) reflects expected optimism. An identified optimism coefficient of 0.008 indicates that the model is less likely to be sample-dependent, suggesting robust performance across different datasets.

We developed a risk stratification tool using easily accessible prognostic factors to support clinical decision-making, particularly in low-and middle-income settings where access to imaging and laboratory tests is limited. The study had some limitations; it would have been more robust if conducted with a prospective design and externally validated. Additionally, future research should refine the model by including key predictors such as income and adherence, which were not captured in the retrospective data collection.

The developed predictive model does not compromise patient safety, as it does not advocate for invasive procedures but rather emphasizes vigilant monitoring and proactive care strategies by guiding clinicians to strictly follow these high-risk patients. Therefore, identifying high-risk patients is crucial, especially in the context of severe acute malnutrition (SAM) in children. By focusing on key predictors such as baseline edema, pneumonia, anemia, and diarrhea, clinicians can prioritize interventions for those most vulnerable to adverse outcomes. The predictive capacity for children with severe acute malnutrition (SAM) who exhibit baseline edema, pneumonia, anemia, and diarrhea is higher. Despite receiving folic acid, Vitamin A supplementation, and intravenous antibiotic treatment, scored 30 on the risk assessment, resulting 0.94 risk probability, which is higher than the 0.6955 Youden index cute of point. This indicates that they are at a relatively high risk for unfavorable treatment outcomes. Such a targeted approach aligns with evidence-based practices, ensuring that healthcare resources are allocated efficiently to those who require the most attention. Ultimately, this strategy aims to improve health outcomes for children suffering from SAM, fostering better recovery and overall well-being.

The nomogram’s clinical benefits were assessed using decision curve analysis (DCA), which compares the advantages of the prediction model against traditional treat-all or treat-none strategies. DCA reveals insights beyond conventional performance metrics like discrimination and calibration. The analysis showed that the nomogram offers a greater net benefit when patient threshold probabilities exceed 21%. For instance, at a personal threshold probability of 30%, the net benefit of using the nomogram for intervention decisions is approximately 0.53, highlighting its significant advantage over alternative strategies. However, its effectiveness declines for threshold probabilities below 21%, indicating reduced utility in those scenarios. Thus, threshold probabilities are crucial in DCA, helping clinicians decide when to apply the nomogram for patients at risk of poor treatment outcomes. This method enables healthcare providers to make informed decisions, optimizing resource allocation and enhancing patient care by focusing on individuals most likely to benefit from targeted interventions. Overall, the nomogram proves to be a valuable tool in clinical practice, improving decision-making in the management of severe acute malnutrition cases and ultimately contributing to better patient outcomes.

The developed risk prediction model for predicting unfavorable treatment outcomes in children with severe acute malnutrition (SAM) is user-friendly and relies on easily obtainable predictors, making it accessible for clinicians at all levels. It allows healthcare providers to assess the risk of poor treatment outcomes and categorize patients as higher or lower risk without requiring complex mathematical calculations. The model is clinically interpretable and validated through decision curve analysis, enhancing its reliability. Notably, this is the first prognostic model focused on unfavorable treatment outcomes among SAM patients in the Amhara Region of Ethiopia, making it particularly relevant to local healthcare settings.

This nomogram serves as a valuable tool for healthcare professionals in personalizing treatment and care for SAM patients. It also supports intensified research and innovation aimed at accelerating the development and implementation of new healthcare tools. Furthermore, policymakers and program managers can utilize this model to design individualized patient-specific policies and programs, ultimately addressing the high rates of poor treatment outcomes in SAM patients. By providing a structured approach to decision-making, the nomogram has the potential to significantly improve patient care and outcomes in the region.

The main strength of this study was robust due to its multi-setting approach, ensuring representativeness and transferability. And also, the constructed nomogram was with sufficient events per parameter and selected using lasso regression. However, limitations include the retrospective design, which may have missed the key predictive variables. Besides, the model was not externally validated using an independent dataset.

### Implication

These study implies that using the model to admit high-risk children in stabilization center yields a greater net benefit than not admitting/providing specialized care for all SAM children, regardless of risk thresholds.

## Conclusion

The developed nomogram serves as a reliable tool for predicting unfavorable treatment outcomes in patients with SAM, demonstrating satisfactory accuracy and good calibration. This model supports clinical decision-making by helping clinicians identify at-risk patients early, thereby reducing the high rates of adverse outcomes associated with SAM. The small optimism coefficient from internal validation indicates minimal overfitting, ensuring the model’s accuracy when applied to new data. Clinically interpretable and validated through decision curve analysis, the nomogram holds significant potential for practical use, particularly in improving patient care amid global political instability and displacement.

Individual patient risk prediction models are essential in modern medicine, particularly for managing SAM. Clinicians should utilize the developed nomogram to identify patients at risk of poor treatment outcomes early, thereby reducing unfavorable results. Patients can leverage the model for informed treatment choices based on their personalized risk. Policy makers and program managers are encouraged to integrate the prediction model into strategies for effective SAM management. Future researchers should enhance the model’s performance by adding new predictors and validating it externally to broaden its applicability in different contexts.

## Data Availability

The original contributions presented in the study are included in the article/Supplementary material, further inquiries can be directed to the corresponding author.
